# The Aerial Guide Dog: A Low-Cognitive-Load Indoor Electronic Travel Aid for Visually Impaired Individuals

**DOI:** 10.3390/s24010297

**Published:** 2024-01-04

**Authors:** Xiaochen Zhang, Ziyi Pan, Ziyang Song, Yang Zhang, Wujing Li, Shiyao Ding

**Affiliations:** Department of Industrial Design, Guangdong University of Technology, Guangzhou 510090, China; xzhang@gdut.edu.cn (X.Z.); 2112217009@mail2.gdut.edu.cn (Z.P.); 2112217004@mail2.gdut.edu.cn (Z.S.); 2112017031@mail2.gdut.edu.cn (Y.Z.); 3119008036@mail2.gdut.edu.cn (W.L.)

**Keywords:** indoor electronic travel aid, visual impairment, wearable assistive devices, cognitive load

## Abstract

Most navigation aids for visually impaired individuals require users to pay close attention and actively understand the instructions or feedback of guidance, which impose considerable cognitive loads in long-term usage. To tackle the issue, this study proposes a cognitive burden-free electronic travel aid for individuals with visual impairments. Utilizing human instinctive compliance in response to external force, we introduce the “Aerial Guide Dog”, a helium balloon aerostat drone designed for indoor guidance, which leverages gentle tugs in real time for directional guidance, ensuring a seamless and intuitive guiding experience. The introduced Aerial Guide Dog has been evaluated in terms of directional guidance and path following in the pilot study, focusing on assessing its accuracy in orientation and the overall performance in navigation. Preliminary results show that the Aerial Guide Dog, utilizing Ultra-Wideband (UWB) spatial positioning and Measurement Unit (IMU) angle sensors, consistently maintained minimal deviation from the targeting direction and designated path, while imposing negligible cognitive burdens on users while completing the guidance tasks.

## 1. Introduction

According to August 2023 statistics from the World Health Organization (WHO), at least 1 billion individuals suffer from vision impairments or blindness. Vision disorders or blindness affect people of all ages, potentially limiting their educational development, reducing labor participation rates, social interaction, and independence, and often lead to a high prevalence of depression. These issues significantly impact the quality of life for individuals with visual impairments [1,2,3,4,5].

Vision is one of humans’ most important senses, essential for normal living and normal moving around. When travelling in unknown environments, it aids individuals in recognizing environmental features to find the correct path and avoid potential hazards on the way [6,7]. However, for individuals with visual impairments, navigating unfamiliar environments and complex buildings is particularly challenging. They often cannot identify key features to guide movements for negotiating stairs/steps/doors or avoid obstacles, such as walls/people/furniture, etc., to reach their desired destination, resulting in feelings of insecurity and anxiety. In fact, up to 70% of individuals with visual impairments tend to avoid moving around independently in indoor spaces, perceiving shopping malls as one of the most challenging environments. When it is essential for them to go shopping, they must rely on getting help from sighted people, which not only undermines their confidence and independence, but also significantly affects their ability to gain more experience in carrying out indoor everyday activities [7,8,9,10,11,12].

Due to the difficulty visual impaired individuals face in recognizing their surroundings, white canes and guide dogs have become preferred solutions because of their simplicity and intuitive nature. However, they have limitations in that they primarily help in identifying objects near the user and are thus mainly suitable for individuals with reasonable confidence and the ability to move around effectively [5,7,13]. The cane relies on tactile feedback transmitted mainly from its tip when swung at ground level, making it difficult to detect obstacles higher up above the swinging range, placing the users in potential hazardous situations [6,10,13]. While guide dogs offer an intuitive and solution, they are limited by an insufficient supply of trained dogs whose lifespan is relatively short (about 6 to 8 years) and high training costs (≈USD 42,000) [5].

Systems designed to enhance the walking autonomy of blind individuals through various technological solutions are generally referred to as Electronic Travel Aids (ETAs). The design of ETAs is particularly filled with challenges [10], because very demanding requirements need to be met, such as real-time guidance, portability, power limitations, suitable interfaces, continuous availability, independence from infrastructure, low-cost solutions, and minimal training. Simultaneously, the system should be easy to use, clear, and user-friendly [5,6,12,13,14]. However, various studies on assistive technology for the blind have primarily focused on object recognition, navigation, and mobility [7], exploring the diverse needs of visually impaired individuals in different activity scenarios. These studies aim to solve context-specific challenges by developing various technological solutions. Currently, no single assistive device has been developed that can be used as extensively and long term in the lives of visually impaired individuals as traditional white canes and guide dogs. Therefore, the focus should be on developing cost-effective, user-friendly long-term solutions able to be used in real-world situations, rather than solely advancing technology [11].

In the past decade of research on indoor ETAs, substituting visual perception via alternative methods has been a mainstream approach [15] stemming from the theory of sensory substitution neuroplasticity. This refers to the capability of the brain to assimilate specific sensory information in alternative ways [12] and requires individuals to consciously integrate their sensory disability with their other functioning senses; e.g., visual impairment can be replaced with auditory and/or tactile senses [6,11].

Based on such sensory substitution approaches, indoor ETAs have been designed relying on methods to generate stimuli that substitute for vision, and users need to learn how to understand the (auditory/tactile) signals to successfully complete activities such as travelling tasks in complex environments. Complexity in understanding the environmental information has been recognized as placing a large cognitive load on the user [15]. Cognitive Load Theory suggests that our working memory is only able to hold a small amount of information at any one time and that instructional methods should avoid overloading it in order to maximize learning [16], and researchers like Giudice et al. have suggested that developers should focus on assisting users in performing specific and necessary tasks, while minimizing the amount of information passed to the user. Indoor ETAs involve utilizing “perceptual and cognitive factors related to processing non-visual information”; however, the bandwidth of non-visual senses such as auditory, tactile, and olfactory is much smaller than that of vision [17]. Hence, balancing the relationship between the minimum and the necessary information becomes crucial [12,18].

The main sensory approaches for replacing visual information are via auditory and tactile methods. Audio methods can be divided into audio description and spatial audio; audio description can provide general guidance but often lacks the detail needed for precise movements [19,20,21,22,23], whereas spatial audio, which links sound source locations to intended directions, is more intuitive for the user. However, spatial audio can interfere with environmental sounds, which can cause hazardous situations to arise [24,25,26,27,28,29,30]. Tactile methods involve vibrotactile and kinesthetic approaches. Vibrotactile feedback uses vibrations to convey environmental information and can be felt on different body parts, but its effectiveness varies due to factors like body part sensitivity and clothing thickness [30,31,32,33,34,35,36,37,38]. Kinesthetic devices use traction force for providing directional cues. For instance, Antolini et al. proposed a method of providing kinesthetic stimulation to users by tilting a flywheel inside the device, allowing users to determine left or right directions based on the sensation of motion simulated by the flywheel, thereby guiding user navigation [39]. Another method includes devices that change shape to provide directional clues [38]. For example, Spiers et al. proposed a cube-like device with a top section that rotates and extends, providing tactile feedback on various finger areas to indicate direction [38,40,41].

Current sensory compensation methods used in indoor ETAs result in a high cognitive load because they require users to consciously exert effort in engaging their other senses to comprehend the environmental information provided to them while moving about. There is a lack of simple solutions able to provide more natural feedback that is easier to comprehend for visually impaired individuals.

**Figure 1:**
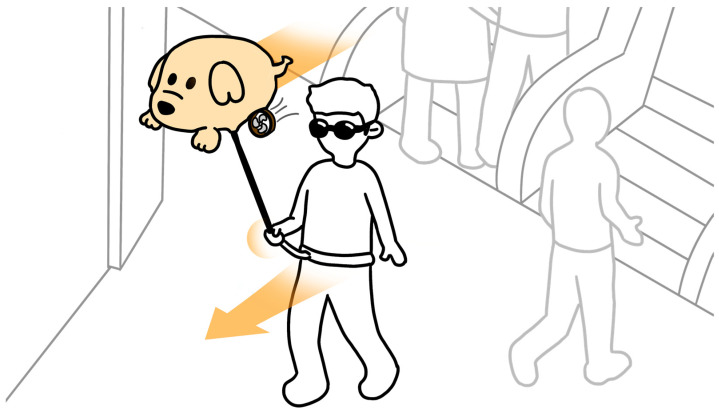
Conceptual diagram of the “Aerial Guide Dog” supporting navigating indoors.

To create an effective and user-centric feedback method for facilitating visually impaired individuals’ independence in performing indoor movements, we introduce an innovative indoor ETA named the “Aerial Guide Dog”, shown in Figure 1. The solution consists of a helium balloon aerostat drone, which is attached to a flexible carbon fiber rod. Visually impaired individuals hold the handle part of the rod and can feel the traction force signals produced by the system to help guide the person to move around safely and reach desired locations. The proposed solution is inspired by the way normal guide dogs provide directional feedback to their master through their leashes while walking. The design aims to recreate this simple and intuitive tactile approach in a more generic manner using robotic drone technology.

The main contributions of this study include:

- An innovative solution for indoor ETAs based on tactile sensory substitution.
- A prototype system that is potentially easy to use, requires short training times, and is cost-effective.
- Preliminary results from pilot studies validating the prototype’s effectiveness through directional perception experiments and path-following tests.

This initial work provides a direction for future research and how a long-term usable indoor navigation assistance system for the visually impaired can be developed.

## 2. Related Work

The preferences, suggestions, and actual needs of visually impaired individuals regarding ETAs in both indoor and outdoor environments are crucial references for researchers developing suitable commercial ETA solutions. In this regard, Plikynas et al. [6] conducted comprehensive interviews with 25 blind experts, revealing that 16 of them avoided using any ETAs for indoor navigation due to the absence of suitable and convenient commercial solutions. Therefore, compared to existing outdoor solutions, the market still demands further development and enhancement of suitable tactile and auditory devices for indoor orientation and navigation [8].

The pros and cons of navigation system feedback methods are a qualitative assessment, varying according to the specific needs and capabilities of users in different environments. Plikynas et al. indicate that, taking voice commands as an example, visually impaired individuals tend to prefer this type of audio feedback for outdoor navigation as compared to indoor environments [6]. Therefore, it is crucial to provide users with appropriate feedback methods tailored to specific situations and needs. Although tactile feedback may encounter limitations in comprehending all transmitted information in areas of perception after prolonged use, visually impaired individuals still show a preference for receiving commands or information through this feedback method in indoor environments [6].Hence, considering tactile feedback as a more accepted method for visually impaired individuals in indoor environments, it should be prioritized as a vital sensory alternative in the development of indoor ETAs.

Enhancing vibration-sensed signals through advanced signal processing algorithms to convey directional information to visually impaired users is a common tactile and effective feedback method. Robert et al. proposed a method called ALVU (Array of Lidars and Vibrotactile Units), which includes a sensor belt worn around the waist and a separate tactile belt worn around the upper abdomen [42]. The sensor belt operates by emitting infrared light pulses to measure the distance between the person and nearby obstacles, effectively detecting obstacles around the individual. In contrast, the tactile belt utilizes vibrating motors to provide feedback. These motors adjust their vibration frequency and intensity based on the distance to detected obstacles, as measured by the sensor belt, thus conveying the distance information of these obstacles to the user [39]. This system has been identified as an effective method of feedback. Khusro et al. developed a real-time feedback system for indoor navigation that utilizes the vibration motors within smartphones to deliver rich tactile information based on vibration characteristics such as frequency, rhythm, and duration. By systematically arranging different lengths of patterns in the manner of Morse code, this system mimics natural tones familiar to users, such as ‘heartbeat’ and ‘knocking’, thereby greatly improving the learnability and understandability of the information received by users [43]. See et al. utilized a robotic operating system to integrate depth camera sensors and obstacle localization algorithms, employing tactile feedback to detect obstacles surrounding the user. This wearable device, equipped with vibration motors in various areas on the user’s body, conveys the location of obstacles by activating the corresponding directional motor and indicates the distance to these obstacles through the intensity of the vibrations. Users can stop and make necessary adjustments based on the specific vibration cues to navigate around all types of obstacles [44].

In most approaches, the tactile signals of the assistive devices developed rely on coding and requiring users to learn to understand the “coded information” corresponding to different vibration signals which can demand significant effort to learn and memorize. Additionally, while the information provided via vibration-based mechanisms is generally effective, prolonged use can lead to fatigue and numbness, resulting in individuals being unable to comprehend all the information for effective use [6]. Another approach using force feedback for guidance has been found to be more intuitive and less demanding cognitively. Federica et al. [10] proposed an ETA system where users receive directional haptic feedback through forces provided by motors worn around in an armband. The device works through the motors spinning in opposite directions to tighten or loosen the armband, advising the user to walk or stop, and the motors spinning in the same direction, causing the armband to slide up or down the arm advising the user to turn left or right. This simple method has been evaluated to convey clear directional information through pressure and skin stretching on specific body parts, akin to a volunteer holding a visually impaired person’s arm for guidance. Navigating by replicating such familiar approaches from the experiences of visual impaired persons is clearly a valid method to adopt in realizing effective user-centered designs that can work well in real-world situations. However, an issue that needs to be addressed is that the thickness of clothing needs to be taken into account as it can affect the user’s perception of the signals. Therefore, compared to reproducing the method of volunteers guiding individuals with visual impairments, the Aerial Guide Dog chooses the more sensitive finger pulp area for tactile feedback. By emulating the working method of guide dogs to lead the visually impaired, it can enhance the effectiveness of perception and reduce the impact of other external factors.

Avila et al. [24] demonstrated that an assistive navigation system with a drone as the guidance module is an efficient and accurate method of guiding, as it provides continuous directional feedback [45]. Notably, the drones developed utilize a soft rope to relay the forces to the user to enhance the independent navigation abilities of visually impaired users. However, due to the use of a soft rope connection, users must maintain a strict relative spatial position with the drone to fully perceive the traction force, as any change in relative position renders it ineffective. When users follow the drone for navigation, changing their walking speed can cause a mismatch between the expected and actual traction forces provided by the drone, leading to ambiguous directional guidance [46]. Compared to the guidance systems of commercial drones, the Aerial Guide Dog utilizes a quieter helium balloon aerostat drone and uses a flexible carbon rod for the traction rope, ensuring that users can clearly perceive directional signals by merely holding the handle. This makes the new approach presented here more in line with the visually impaired users’ requirements as well as being cost-effective [7,11].

Compared to the traditional robotic guide dog method developed by Hwang et al., the advantage of the Aerial Guide Dog lies in its flying guidance approach [47], which reduces ground interaction challenges with the environment encountered [7]. Being above the ground, it also has a wider field of view, thereby improving its range to provide more complete environmental information to the user. Furthermore, this aerial approach reduces the wear and tear often seen in ground-based systems due to continuous contact with irregularities of the ground surface.

The introduction of the Aerial Guide Dog as an indoor ETA is felt to represent a significant advancement in technology to help individuals with visual impairments move around effectively. Furthermore, it underscores the importance and practical applicability of the Aerial Guide Dog’s tactile sensory substitution approach, which needs to be investigated in future research on indoor ETAs.

## 3. Design and Implementation

### 3.1. Spatial Mapping and Perceptual Characteristics of the Index Finger

**Figure 2:**
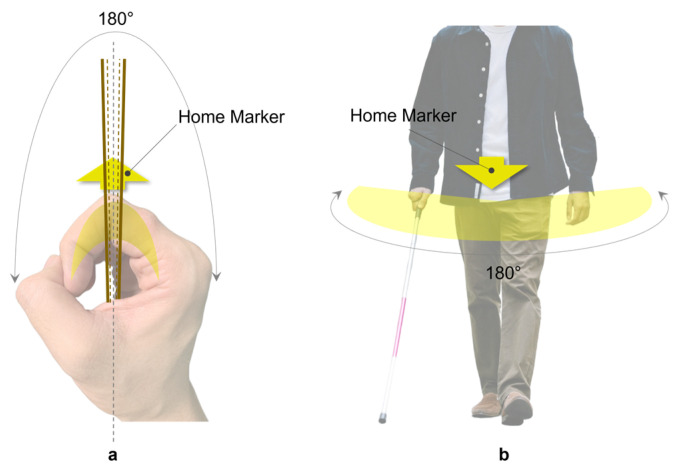
(**a**) Spatial mapping of the index finger with the area directly in front of the user; (**b**) normal movable directions in space.

The study introduces an interaction method aimed at simplifying navigation tasks by centering on the right hand, specifically the index finger as the tactile perception area, to establish a body-based spatial reference system that leverages human innate proprioception to reduce potential cognitive load [48]. The specific implementation details, as shown in Figure 2a, show the right hand in a gripping position, placed in front of the body, holding the assistive handle of the Aerial Guide Dog, and maintaining the spatial relationship of the right hand relative to the body. When the Aerial Guide Dog conveys directional information to the right index finger, the wrist must rotate in the prompted direction, allowing directional information to be obtained by perceiving the change in the right wrist’s position relative to the body.

The spatial reference system utilizes human proprioception, which mainly includes joint position sense and joint static awareness, playing a crucial role in understanding spatial environments. Joint position sense is used to ensure that users are aware of the relative position between their right index finger and their body, while joint static awareness ensures that users can determine this positional relationship even when stationary. This inherent bodily spatial positioning ability enables users to instinctively grasp the positioning and interconnection of limb joints relative to the entire body [49]. Furthermore, considering the successful performance of past projects, assistive devices for the blind that use proprioceptive correspondence as a fundamental element can provide a more natural method of orientation interaction [12].

This method involves creating a spatial positioning system centered around the index finger: the interval from the second joint to the fingertip corresponds to the spatial mapping of the 90° to 0° area directly in front of the user, while the interval from the second joint to the base of the finger corresponds to the 90° to 180° area in front of the user, as illustrated in Figure 2. Based on this, users can obtain directional information within the 0° to 180° area directly in front of them.

Additionally, the finger tips and fleshy inside parts of the fingers are the most densely populated area for sensory organs, with five types of receptors distributed beneath the finger pads, including Ruffini corpuscles, Meissner corpuscles, Pacinian corpuscles, Merkel discs, and free nerve endings. Therefore, using the these areas of the fingers as the receptive part results in clearer perception of tactile information [48]. Moreover, the continuous traction force stimulation transmitted to the fingers during the use of the Aerial Guide Dog for indoor navigation falls under the kinesthetic category [45,50], where the effectiveness of guidance based on continuous traction force feedback is primarily attributed to Ruffini corpuscles as the receptors for traction force stimulation [51]. The slow-adapting nature of Ruffini corpuscles ensures that the sensation of the traction force does not diminish immediately when the stimulus is continuously applied [47,48,49] so that users can continuously perceive the transmitted traction force stimuli and its directional information through the fingers, thereby ensuring the effectiveness of continuous navigation.

It is felt that this approach simplifies cognitive processing by creating an angular mapping between the joint of the index finger and the user’s frontal area, using the index finger pulp as the tactile receptive area for directly receiving directional signals, which reduces the cognitive effort required to understand and interpret the navigation signals [19,52]. 

### 3.2. Wearable Tactile Prototype and Its Interaction Methodology

To implement traction force feedback, we utilize servo motors and a helium balloon aerostat drone to build a simple, intuitive, and low-cost prototype design to validate the feasibility of mapping traction force directional signals to a spatial reference system established based on the user’s body.

**Figure 3:**
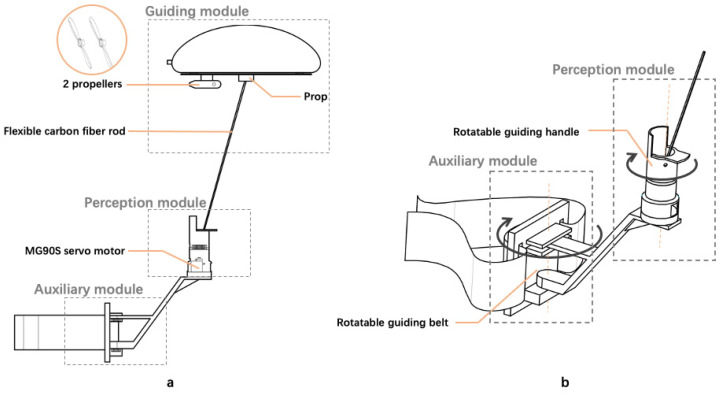
Schematic diagram of the Aerial Guide Dog interactive prototype structure: (**a**) overall schematic of prototype, encompassing the guidance module, the perception module, and the assistive module; (**b**) the perception module assists in wrist rotation, and the auxiliary module aids in body rotation.

The prototype system developed comprises three main modules:

- The guidance module: as shown in Figure 3a, the main component is a 32-inch aluminum film balloon filled with helium (here referred to as the helium balloon aerostat drone). A 3D-printed prop acts as a connecting element, securing the helium balloon aerostat drone to a flexible carbon fiber rod. The bottom of the helium balloon aerostat drone is also equipped with 2 propellers, which generate horizontal thrust when rotating, propelling the aerostat drone forward.
- The perception module: as shown in Figure 3a, the MG90S servo motor inside the perception module is connected to the flexible carbon fiber rod, controlling its angular position to convey the direction of movement to the user’s fingers. As depicted in Figure 3b, the exterior of the perception module features a rotatable 3D-printed handle, which the user grips to assist in adjusting wrist rotation. Additionally, the handle is embedded with a vibration motor, providing extra tactile feedback for each movement of the rod.
- The auxiliary module: as shown in Figure 3b, the auxiliary module primarily consists of a servo motor, a 3D-printed support structure, and a waist belt. It is designed to assist users in adjusting their body orientation.

To implement traction force feedback, we utilize servo motors and a helium balloon aerostat drone to construct a simple, intuitive, and low-cost prototype design to validate the feasibility of mapping traction force directional signals to a spatial reference system established based on the user’s body.

**Figure 4:**
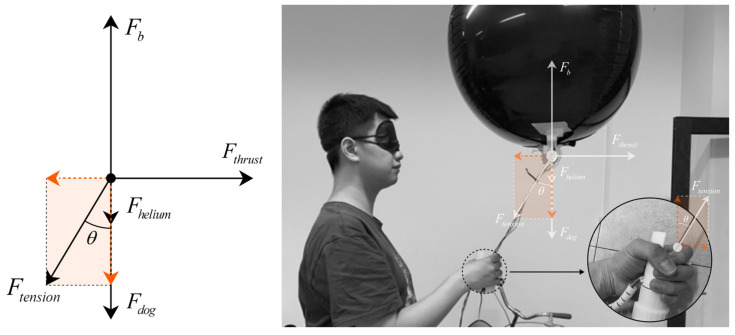
Schematic diagram of the force analysis of the Aerial Guide Dog during operation.

The overall force analysis of the Aerial Guide Dog prototype during operation is shown in Figure 4 through the upward buoyant force $F_{b}=\rho_{air}Vg$, where $\rho_{air}=1.25\;{\mathrm{kg}/m}^{3},V,g=9.8\;{m/s}^{2}$ are the density of air, the volume of the balloon, and the acceleration of gravity, respectively. 

Apparently, the downward gravity composed of the weight of the Aerial Guide Dog including the weight of helium can be expressed as $F_{down}=F_{dog}+F_{helium}$.

Thus, given the overall status of floating or hovering in the air, the balance between buoyancy and downward gravity can be expressed as $F_{b}=F_{down}$.

Given the density of helium $\rho_{he}=0.178\;{\mathrm{kg}/m}^{3}$, it is not hard to find that $F_{dog}=(\rho_{air}-\rho_{he})Vg\approx Vg$.

In reality, by inflating more helium in the balloon, the Vg is slightly greater than $F_{dog}$, causing a tender upward force on the rod where the other end is held by human hands.

A thrust force $F_{thrust}$ can be generated by the dual motors to drive the Aerial Guide Dog towards different directions. Consequently, the force is transmitted via the rod for the user to follow. 

Ideally, when the Aerial Guide Dog is moving horizontally and the rod is at an angle θ with respect to the vertical direction, the tension in the string can be decomposed into two components, $F_{tensionV}=F_{tension}\cos(\theta )$ and $F_{tensionH}=F_{tension}\sin(\theta )$, where the former is the vertical component counterbalanced by the buoyancy and weight of the balloon and the latter is the horizontal component that the user feels as a traction force.

The magnitude of this force will depend on the difference between the thrust and the drag, as well as the angle of the string. Assuming a steady-state motion where acceleration is zero (constant velocity), the net force in the horizontal direction is zero, and thus $F_{thurst}-F_{tensionH}=0$, where we simplify the air resistance as zero given the fact that the speed of the proposed Aerial Guide Dog is slow.

Further, we obtain $F_{tension}=\frac{F_{thurst}}{\sin(\theta )}$. In application, factors like the flexible carbon rod, dynamic changes in the Aerial Guide Dog speed, and the user’s movements will complicate this model.

The guidance system of the entire interaction prototype can be compared to the working principle of a normal guide dog. In the guidance module, the helium balloon aerostat drone, akin to a guide dog, is connected to the flexible carbon fiber rod, which acts like the dog’s harness, transmitting traction forces directly. The end of the flexible carbon fiber rod is attached to the servo motor of a rotatable guiding handle within the perception module, generating directional signals similar to those produced when a guide dog turns. The user grasps the handle, with the index finger pulp touching the flexible carbon fiber rod, thereby feeling the traction stimulus transmitted from the handle and understanding its directional information. Additionally, the auxiliary module, equipped on the user’s waist and integrated with the perception module, aids in aligning the direction of the user’s body and the second joint of the index finger. This alignment responds to the change in the direction indication signaled by the carbon fiber rod in the guidance module, thereby enhancing precise navigational guidance.

**Figure 5:**
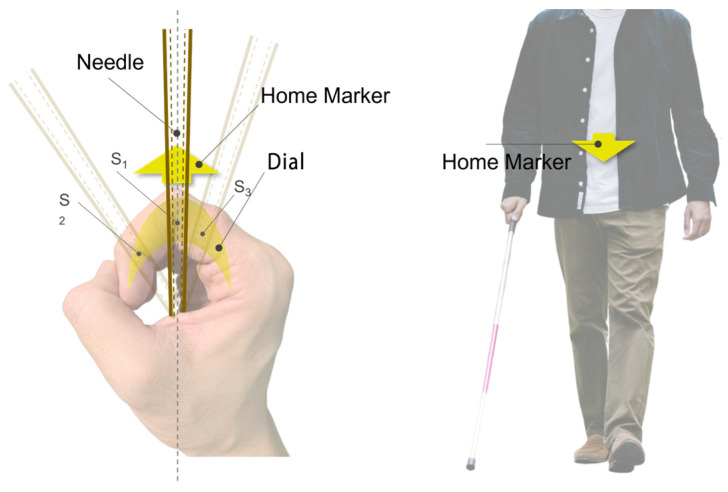
Using the index finger as a compass-style spatial positioning system; S1, S2, and S3 represent the contact points on the index finger’s tactile receptive area when the tactile needle is directed towards different target directions.

Specifically, as shown in Figure 5, the flexible carbon fiber rod acts as a tactile rod, transmitting the traction force directional signals to the receptive area of the index finger. The user holds the assistive guiding handle of the perception module, orienting the second joint of the index finger directly forward, with the user’s fist serving as the central element of the spatial framework. Based on this, the user’s fist and the flexible carbon fiber rod create a directional feedback mechanism similar to that of a compass. Within this spatial framework, the direction indicated by the second joint of the index finger is referred to as the “Home Marker”, functioning similarly to the direction-of-travel arrow of a compass. The user needs to align their body’s forward direction with this marker.

**Figure 6:**
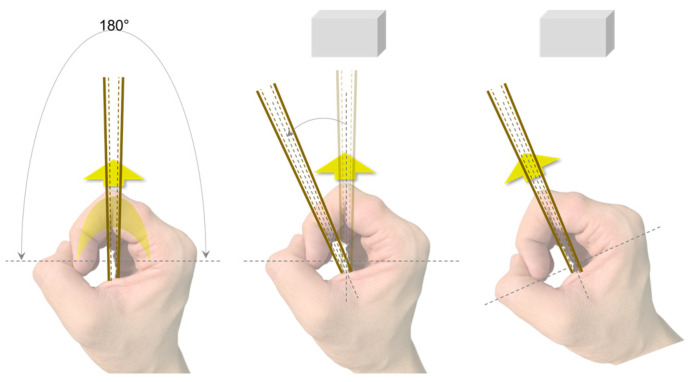
Schematic diagram of the interaction gesture.

Utilizing proprioception feedback for this compass-style interaction method is more consistent with the user’s inherent cognitive processing mechanisms, simplifying navigation tasks and allowing the user to navigate without focusing on the precise angular deflection of the tactile needle. Instead, the user only needs to subjectively judge the position of the contact point between the tactile rod and the index finger relative to the Home Marker, ensuring the rod remains aligned with the Home Marker during rotation. As illustrated in Figure 6, in the initial state, the tactile needle’s indicated direction and the Home Marker are aligned with the user’s forward direction. When the Aerial Guide Dog transmits angular information, the tactile needle deviates from the Home Marker, rotating towards the target direction. At this point, the user’s index finger feels the tactile angular offset so that the user can rotate their wrist to realign the Home Marker with the new target direction and adjust the body orientation using the auxiliary module until the user’s forward direction aligns with the Home Marker, achieving the alignment of the forward direction and the target direction. This navigation method is simple and intuitive, requiring little cognitive thinking. Moreover, the device’s continuous kinesthetic feedback ensures that the user dynamically tracks the position of the contact point between the feedback signal and the user’s index finger throughout the process.

Additionally, considering the hardware limitations of the MG90S servo motor, the range of motion for the tactile needle is restricted to ±90° (a 180° area directly in front of the user). However, for practical use, guidance assistance devices need to provide feedback across a 360° range. To meet this requirement, when users perform rotation tasks beyond the ±90° range, the assistive guidance handle will first send a special directional signal to the user: the tactile needle will rotate to the target direction’s 90° position and immediately reverse back to the initial position. This special signal alerts the user to execute a 90° rotation. Therefore, when users need to rotate beyond the restricted angle, the assistive guidance handle will initially convey a special signal to prompt a 90° rotation. If the angle for the subsequent rotation lies within the motor’s operational range, a regular directional signal is then issued, guiding the user to follow the direction indicated by the tactile needle.

This improvement ensures comprehensive coverage of the feedback range, matching the device’s capabilities with comprehensive indoor navigation support requirements. Subsequent experimental analyses will evaluate two types of rotation situations separately: a special direction signal (SDS) situation, involving special directional signals for user rotations exceeding ±90°, and a non-special direction signal (non-SDS) situation, involving user rotations within the ±90° range. A subsequent experiment distinguishes these two situations to show how human rotating behaviors affect the angles of deviation.

### 3.3. Guidance Strategy

#### 3.3.1. Direction Indication Strategy

The directional indication strategy is derived from the interactive method proposed in this study, aiming at providing directional guidance for visually impaired users during navigation. In this strategy, the user’s index finger is considered the dial of a tactile compass. When correctly holding the assistive guiding handle, the position of the second joint of the index finger is marked as 0°. Directions to the right of this reference point are positive, and those to the left are negative. As in Equation (1), in this strategy, *θt* represents the angle of the tactile pointer, corresponding to the target direction; *θc* represents the angle of the Home Marker, which is the direction indicated by the second joint of the user’s index finger, and the user should ensure that their forward direction corresponds with it; *θg* represents the angle that the user needs to adjust. Specifically, when *θg* = 0, it means that the angle of the tactile pointer is aligned with the Home Marker, indicating that the user is in the correct forward direction. When *θg* is positive, it implies that the tactile pointer is to the right of the Home Marker, suggesting that the user should turn clockwise. Conversely, when *θg* is negative, it indicates that the tactile pointer is to the left of the Home Marker, suggesting that the user should turn counterclockwise.

*θg* = *θt* − *θc*, (1)

Therefore, users can determine the guiding direction simply by discerning the position of the contact point between the tactile needle and the index finger relative to the second joint of the index finger, and adjust their own direction with the help of the auxiliary module.

#### 3.3.2. Path-Following Strategy

**Figure 7:**
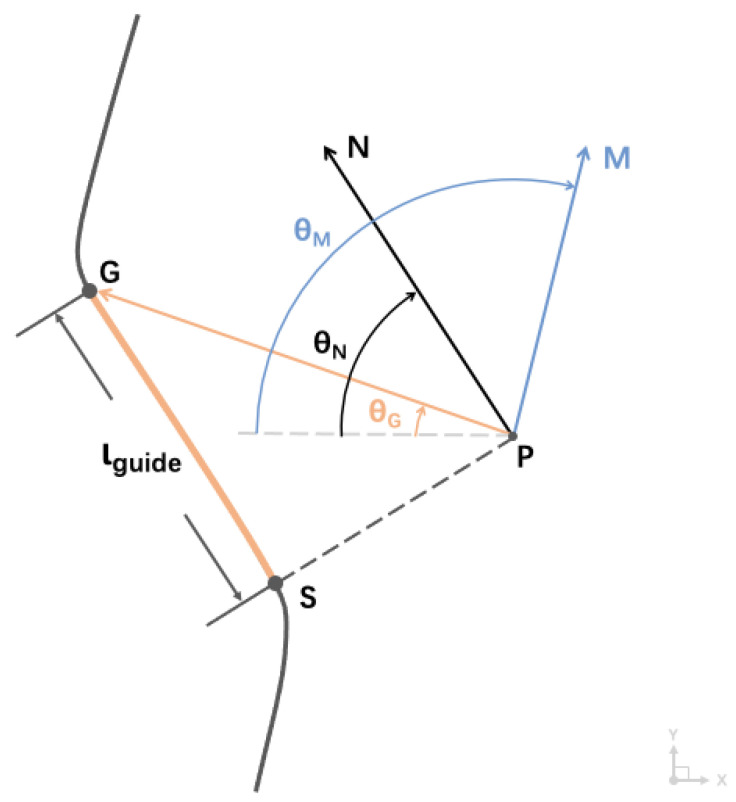
Visualization of the path-following strategy.

To enable users to perform real-time navigation and directional decision making on designated paths, the direction indication strategy outlined in Equation (1) was developed. Additionally, the *Codes for Accessibility Design* specifies an indoor walkway and doorway width range of 80 cm to 180 cm [53]. Considering factors such as foot traffic, the width of the experimental path, λ, was defined as 60 cm. Referring to the navigation strategies outlined in [19], as shown in Figure 7, point P represents the user’s current position, and point S represents the point on the path’s central line closest to the user. The shortest distance from the user to the central line is represented by |PS|. *θ_M_* represents the user’s current actual direction of motion, with its corresponding planar vector being $\underset{PM}{\to }$. Point G, which is a constant guiding distance ι_guide_ ahead of point P on the central line, corresponds to the target direction point. Therefore, the line PG represents the target direction, with its corresponding planar vector represented by $\underset{PG}{\to }$. The user can move in the direction *θ_G_* to realign with the central line. When |PS| > 30 cm, it indicates that the user has moved outside the path boundaries. In this case, the Aerial Guide Dog sends a directional signal *θ_G_*, guiding the user back within the established path boundaries.

This path-following strategy aims to keep the user in line with the path’s center, yet this exact consistency deviates from common walking habits. Therefore, these uncorrected deviations lead to the auxiliary guiding handle providing high-intensity directional signals, resulting in an intrusive experience for the user [19]. To mitigate this negative impact on the user’s indoor navigation experience, it is necessary to adjust the path-following strategy to reduce the intensity of directional signals, thus enhancing the user experience while maintaining efficient guidance.

Therefore, the planar vector $\underset{PN}{\to }$ is introduced, which remains parallel to the path’s central line, with the corresponding angle being *θ_N_*. Based on this, *θ_G_* should satisfy the following relationship (2):

0° < |*θ_N_ − θ_G_* | < 15°, (2)

After adjustment, when the user’s deviation from the target direction is within ±15°, they will not receive directional cues from the Aerial Guide Dog, alleviating the negative impact of the intrusive experience.

## 4. Pilot Study

### 4.1. Directional Perception Study

#### 4.1.1. Participants and Apparatus

A directional perception experiment was conducted to systematically evaluate participants’ directional accuracy in a controlled environment. The study involved 16 participants, including 10 males and 6 females, ranging in age from 23 to 26 years. All participants, possessing normal visual abilities, were required to wear a blindfold throughout the experiment.

**Figure 8:**
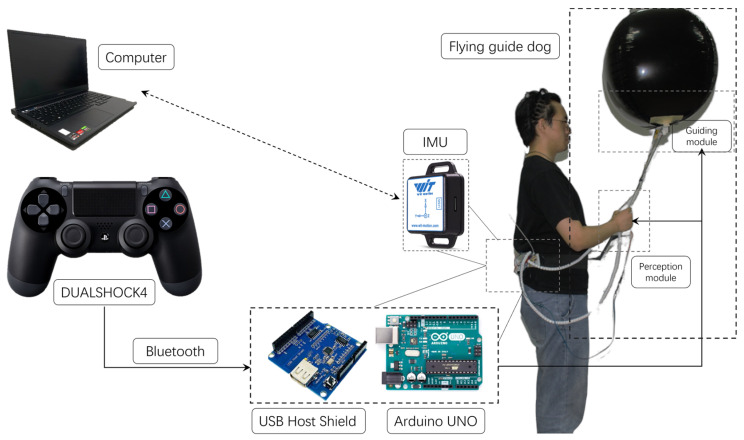
Apparatus and experimental setup.

The experiment was conducted in an indoor space measuring 5.7 × 3.6 m. At the heart of the experimental equipment is the BWT61CL model Inertial Measurement Unit (IMU), a common positioning mechanism responsible for monitoring the directional changes of participants before and after receiving directional feedback [54]. Despite the presence of cumulative errors, its accuracy in estimating the position and body orientation of participants can be enhanced by refining directional errors [6], ensuring a measurement precision within 0.5° on the Z-axis. As shown in Figure 8, the device communicates with a PC server through a Bluetooth 2.0 USB-HID adapter, transmitting real-time directional data at a rate of 100 Hz.

In the experiment, a DUALSHOCK4 controller (Sony, Tokyo, Japan) is connected to a low-cost microcontroller, Arduino UNO (Arduino, Italy) to transmit directional signals. The signals trigger the servo motor and vibrator inside the assistive guiding handle of the perceive module to provide participants with directional cues for traction stimulus. Upon receiving these signals, participants adjust their direction accordingly. Then, the IMU captures these position changes and transmits the data to a PC for further analysis.

#### 4.1.2. Procedure

Before the formal experiment began, participants were introduced to the purpose and basic procedure of the directional perception experiment and then given specific instructions on how to use the Aerial Guide Dog prototype. Initially, participants, with the help of researchers, fitted the auxiliary module around their waists and held the assistive guiding handle, using the pulp of their index finger to feel the traction force stimuli applied by the tactile needle. Emphasis was placed on distinguishing the direction of deviation between the contact point of tractive force stimulation and the second joint of the index finger. Then, participants learned how to use the auxiliary and perception modules to adjust body and wrist turning. After completing the learning phase, participants proceeded to the experimental site equipped with the IMU, where they had to complete six randomized directional perception tasks to familiarize themselves with the interaction system. This step-by-step approach ensured that participants were thoroughly prepared before the official start of the experiment.

At the start of the experiment, participants stood still for IMU calibration. Tasks were categorized into two groups at 30° intervals for comprehensive navigation scenario coverage: Group 1 included 180°, ±150°, ±120°, ±90°, ±60°, and ±30°; Group 2 included ±165°, ±135°, ±105°, ±75°, ±45°, and ±15°. It is important to note that angle commands beyond ±90° triggered special directional signals, and data were gathered for situations with and without these signals. Participants adjusted their body orientation to align the Home Marker with the tactile needle upon receiving commands. Upon completion, participants reported back to the control center, and then the tactile needle reset to its starting position, followed by a one-second pause before the next task commenced. The system logged the time taken and angular deviation for each participant’s task completion. Throughout, the experiment prioritized participant safety and adhered to strict ethical standards.

#### 4.1.3. Results

To preliminarily assess the impact of human factors, Table 1 presents the data quality from the directional perception experiment conducted by 16 participants, including the proportion of valid data for angular deviation and task completion time under two situations: SDS situations and non-SDS situations. SDS situations encompass complete data for all angle tasks, while non-SDS situations include data excluding the autonomous 90° rotations by participants in SDS situations.

According to Table 1, it can be observed that the proportion of valid data between SDS situations and non-SDS situations is remarkably similar across gender and group categories. Specifically, the average angular deviation in non-SDS situations is 8.84° (SD = 6.08) with an average task completion time of 4.15 s (SD = 2.11). Additionally, in SDS situations, the average angular deviation is 9.71° (SD = 6.12), and the average task completion time is 7.37 s (SD = 5.07).

Since the measurement results of the directional perception experiment were not normally distributed, non-parametric tests were used for analysis. Specifically, the Wilcoxon signed-rank test was applied to two paired samples [55] to analyze whether the introduction of different task groups, different turning directions, and special direction signals had a statistically significant impact on angular deviation.

The test results, as shown in Table 2, indicated no significant statistical difference in angle deviation between different task groups. As presented in Table 3, there was no significant statistical difference in angle deviation between different turning directions. Furthermore, as shown in Table 4, the introduction of special direction signals did not significantly affect angular deviation. This suggests that incorporating a special signal for a natural 90-degree human turn in the guidance strategy is a reasonable and effective navigation strategy that aligns with human spatial mapping and does not impact navigation accuracy.

### 4.2. Evaluation of Path-Following Performance

#### 4.2.1. Participants and Apparatus

The path-following experiment additionally recruited 10 participants, comprising 7 males and 3 females aged between 23 and 26 years. The experiment, based on the equipment and system of the directional perception experiment, utilized Ultra-Wideband (UWB) technology for positioning and orientation. With a working range of 90 m in low data transmission mode, UWB sensors are particularly suited for deployment in large structures, making this technology a potential top choice for maximizing accuracy from a technical perspective [6]. The experiment recorded the participants’ movements on a two-dimensional plane, subsequently transferring the collected data to a PC for further analysis. In this evaluation, we applied the Wizard of Oz method, which allows a well-trained researcher to manually control the Aerial Guide Dog to follow the pre-designed path. The reason we applied the Wizard of Oz method is that unexpected incidents always occur, especially since the Aerial Guide Dog is easily deviated due to the user’s reaction force, and its driving also requires high skills. Accordingly, we conducted rigorous training with the researchers to ensure that the response time for navigation strategies was within 100 ms, in order to meet the requirements of conducting experiments using the Wizard of Oz method.

#### 4.2.2. Experimental Path 

**Figure 9:**
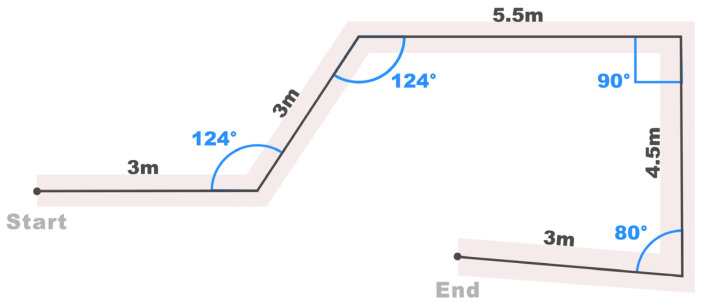
The experimental path incorporating 4 types of daily indoor travel section scenarios.

Drawing inspiration from typical daily indoor travel scenarios, we designed a coherent path in our path-following experiment, incorporating segments such as Straight Path (SP), Right-angle Turn (RT), Acute-angle Turn (AT), and Obtuse-angle Turn (OT), as illustrated in Figure 9. These path segments, while not encompassing all possible scenarios, effectively represent a majority of situations encountered in daily activities. The specifications of this coherent path are as follows: a total length of 19 m, with RT angles at 90°, AT angles at 80°, and OT angles at 124°. Following the path-following strategy discussed in Section 3.3.2, the path’s width was set to 60 cm. 

#### 4.2.3. Procedure

Before initiating the formal experimental procedure, participants were systematically provided with comprehensive instructions and guidance: the activation and deactivation of helium balloon aerostat drone, respectively, signaled the participant to start and stop moving. The thrust generated by the activated drone was converted into directional traction force feedback through the tactile needle, offering directional guidance to the participants. On the other hand, deactivating the helium balloon aerostat drone and the tactile needle’s deviation from the index finger’s navigational range served as signals for participants to stop navigation, adjust their direction, and then continue moving forward. 

Subsequently, researchers guided participants along the designated path. After this, participants were given the option to further learn the system’s usage and various interaction instructions. The guidance phase ensured that participants became familiar with the system’s functionalities and navigation interaction. Additionally, for precise tracking of body orientation, an Inertial Measurement Unit (IMU) was affixed near the participant’s navel area. 

During the formal experiment, if a participant was about to step outside the 60 cm path boundary, the system would immediately issue a stop signal. The participant would then readjust their direction based on the guidance of the tactile needle, returning within the path area. During the experiment, we encouraged participants to express their feelings at any time. This experiment strictly adhered to safety protocols to safeguard participant health, and all procedures underwent ethical compliance review.

#### 4.2.4. Result

The data collected from the experiment showed that all participants successfully completed the path-following task within 3 min. Specifically, the average time taken by all participants to complete the task was 105.24 s (SD = 26.46). The shortest time to complete the task was 73 s, and the longest was 145 s.

**Figure 10:**
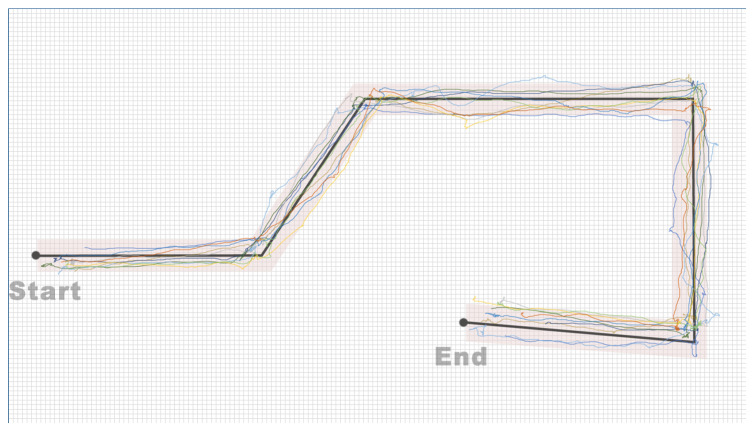
Movement trajectories of 10 participants on designated path in path-following performance evaluation study, distinguished by color.

Figure 10 displays the movement trajectories of 10 participants on the designated path, with colors distinguishing each participant’s trajectory. The recorded trajectories show that, with the navigational support of the Aerial Guide Dog, all participants smoothly passed through the designated path 60 cm wide.

Therefore, the pilot study preliminarily validates the effectiveness of the Aerial Guide Dog in guiding individuals with visual impairments in indoor navigation through traction force feedback, covering both basic directional perception tasks and more complex path-following challenges.

## 5. Discussion

This study developed and preliminarily evaluated a tactile sensory substitution method and prototype for indoor navigation based on traction force perception. It aimed to minimize the cognitive load of individuals with visual impairments during indoor navigation to enhance user experience. Furthermore, the effectiveness of this method in improving directional perception and path-following tasks for individuals with visual impairments was preliminarily assessed using the interaction prototype.

In this study assessing the accuracy of directional perception when users employ the Aerial Guide Dog, a pilot experiment was conducted with 16 blindfolded participants. Additionally, further data analysis was carried out to explore whether “tasks in different perceptual areas of the index finger have an impact on angular deviation”.

**Figure 11:**
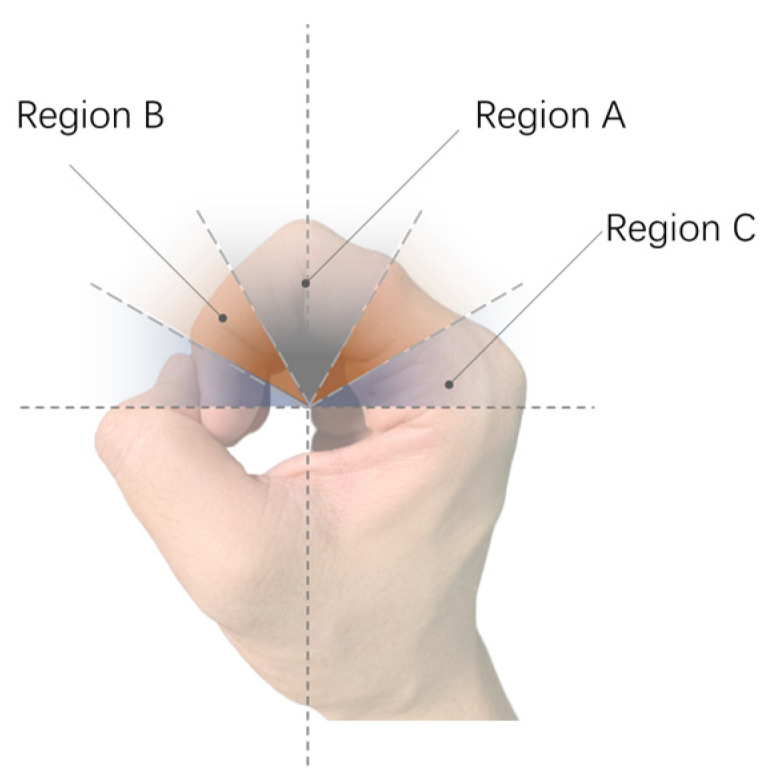
Schematic diagram of the division of the receptive area of the index finger.

The receptive areas of the index finger were divided based on the deviation angle of the tactile needle from the Home Marker. As shown in Figure 11, the area close to the Home Marker is defined as Area A, corresponding to a target angle range of ±30°, representing minor turns. The area near the base of the finger is defined as Area C, corresponding to a target angle range of ±(75°~90°), representing more extreme turning scenarios in indoor guidance; all other areas are collectively referred to as Area B. Based on this division of the index finger areas, the angular deviation data under the non-SDS situation was divided into three groups of samples and analyzed using the Friedman test.

**Figure 12:**
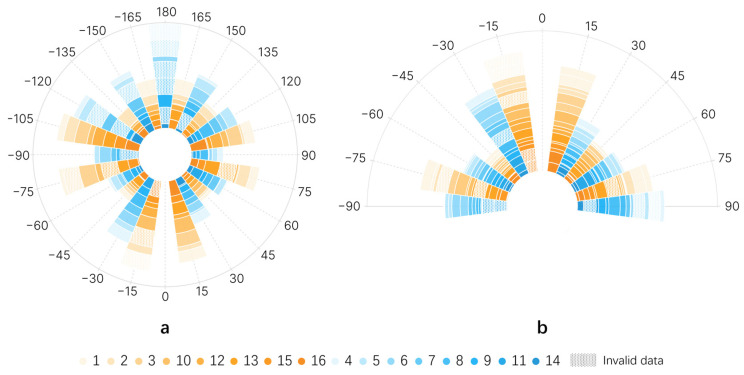
Results of the directional perception study. Different colors represent the magnitude of deviation values of the experimental data from the target angle for different participants under different orientations. (**a**) Angular deviation in SDS situation. (**b**) Angular deviation in non-SDS situation.

The description of angular deviation data under different finger receptive areas is shown in Table 5, and the test results are presented in Table 6. The visualization of angular deviation results under SDS situations and non-SDS situations is depicted in Figure 12, where different colors represent different participants. The width of the color indicates the magnitude of the deviation, with a larger radius of the graph indicating a larger angular deviation in that directional perception task and, correspondingly, lower navigation accuracy. The results in Table 6 show that the deviation in Area B is significantly smaller than in Area A, with an adjusted significance level of <0.001, and no significant difference compared to Area C. However, the difference between Areas A and C is not significant after adjustment. Additionally, combining Table 5 and Figure 12b, it can be observed that users handled directional signals in Area B best, followed by Area C, and lastly Area A. Furthermore, as shown in Figure 12a, the 180° rotation task had the highest deviation. This task is comprised of two SDS, requiring the user to perform continuous 90° rotations. Therefore, despite lower deviations in Area C compared to Area A, cumulative errors due to human factors can significantly reduce rotation accuracy. This emphasizes the necessity of combining machine-assisted rotation with natural human spatial mapping-based rotation.

Additionally, in the path-following experiment, all participants smoothly navigated the designated path, yet there was a significant difference in task completion time (SD = 26.46). A thorough analysis of the participants’ movement trajectories (as shown in Figure 10), considering observations made during the experiment and a review of the video recordings, revealed a discrepancy between participants’ actual forward direction after adjusting their body orientation and the target direction. This discrepancy partly relates to individual walking styles. Specifically, participants with a splayed-foot walking style needed more frequent adjustments in direction, leading to longer time taken to complete the path. 

At the beginning of the direction perception and path-following studies, some participants indicated they had difficulty in learning how to use the interaction method. However, as the experiment progressed, they gradually adapted to this navigation method using their bodies as spatial reference systems and were able to navigate smoothly based on the traction force direction provided by the Aerial Guide Dog. Although participants expressed a negative attitude towards the interaction method at the start, by the end of the experiments, they had changed their attitude, finding the navigation information conveyed by the Aerial Guide Dog to be relatively intuitive and user-friendly. The inconsistency in the participants’ attitudes before and after the experiments might be attributed to our requirement for them to navigate following specific steps during the interaction. To accurately turn towards the target direction, participants had to first rotate their wrists to align their hands with the target direction and then rotate their bodies to align their fronts with the target. This interaction method broke down the process of using a guide dog for navigation cues into two steps, dissecting a continuous action, which initially caused confusion and required time for understanding and practice. Despite these obstacles, all participants completely mastered this interaction method within 15 min.

## 6. Conclusions and Future Work

This study designed and introduced the Aerial Guide Dog, grounded in the theory of sensory substitution, to guide individuals with visual impairments in indoor navigation. By following traction force, this approach minimizes cognitive load, embodying user-centered principles and emulating the intuitive guiding method of traditional guide dogs. The Aerial Guide Dog, functioning through a helium balloon aerostat drone, allows users to perceive tactile directional information by holding a flexible carbon fiber rod connected to the drone, facilitating intuitive interaction through human instinctive compliance with external force. This innovative approach has been evaluated through a pilot study assessing directional perception and path-following performance, with preliminary results affirming the effectiveness of this indoor Electronic Travel Aid (ETA).

Notably, the “Aerial Guide Dog” distinguishes itself from existing navigation aids in two significant ways. First, it utilizes drag force for guidance, a universally intuitive method requiring less cognitive effort, especially beneficial for long-term usage compared to the active interpretation needed for cues like vibrations or audio signals. Second, its unique design as a helium balloon drone offers the advantage of aerial navigation, effectively overcoming indoor terrain variations and extending operational longevity due to minimal energy expenditure on counteracting gravity. These features highlight the potential of the Aerial Guide Dog as a more accessible, user-friendly navigation solution.

However, we also noted technical limitations that posed challenges, particularly during the initial phases of interaction. These challenges underscore the need for continued refinement of the prototype to enhance its adaptation to natural human responses to traction force guidance. Future research will focus on improving the prototype, with broader testing planned to optimize its functionality and user experience in diverse indoor environments.

## Figures and Tables

**Table 1:** The data quality of the directional perception experiment involving 16 participants.

No.	Gender	Data Group	Proportion of Valid Data (%)	
			SDS Situation	Non-SDS Situation
1	M	Group 1	11/12 (91.67%)	10/12 (83.33%)
2	M	Group 1	11/12 (91.67%)	11/12 (91.67%)
3	M	Group 1	11/12 (91.67%)	11/12 (91.67%)
4	F	Group 2	10/11 (90.91%)	10/11 (90.91%)
5	F	Group 2	10/11 (90.91%)	10/11 (90.91%)
6	M	Group 2	9/11 (81.82%)	11/11 (100.00%)
7	M	Group 2	10/11 (90.91%)	11/11 (100.00%)
8	F	Group 2	9/11 (81.82%)	10/11 (90.91%)
9	F	Group 2	11/11 (100.00%)	11/11 (100.00%)
10	M	Group 1	10/12 (83.33%)	10/12 (83.33%)
11	M	Group 2	8/11 (72.73%)	9/11 (81.82%)
12	F	Group 1	10/12 (83.33%)	10/12 (83.33%)
13	M	Group 1	12/12 (100.00%)	12/12 (100.00%)
14	M	Group 2	11/11 (100.00%)	11/11 (100.00%)
15	M	Group 1	12/12 (100.00%)	12/12 (100.00%)
16	F	Group 1	11/12 (91.67%)	12/12 (100.00%)

**Table 2:** Significance analysis of angular deviation across different task groups. Deviation values are significant at *p* ≤ 0.05.

	M (p25~p75)	Z	*p*
Group 1	8.551 (4.932~15.575)	−0.763	0.445
Group 2	8.686 (5.057~12.765)		

**Table 3:** Significance analysis of angular deviation in different turning directions. Deviation values are significant at *p* ≤ 0.05.

	M (p25~p75)	Z	*p*
Turn counterclockwise	8.108 (4.296~12.162)	−0.352 ^1^	0.725
Turn clockwise	8.004 (4.349~12.175)		

^1^ Based on negative ranks.

**Table 4:** Significance analysis of angular deviation with the introduction of SDS (special direction signals). Deviation values are significant at *p* ≤ 0.05.

	M (p25~p75)	Z	*p*
Non-SDS-Situation	9.014 (4.749~12.785)	−0.591 ^1^	0.445
SDS-Situation	8.623 (5.654~15.170)		

^1^ Based on negative ranks.

**Table 5:** Average angles and Chi-square analysis results for different finger receptive areas in non-SDS situation.

	M (p25~p75)	Chi-Square
Region A	10.693 (7.947~17.224)	14.533
Region B	5.427 (1.848~8.558)	
Region C	7.614 (3.474~12.694)	

**Table 6:** Significance comparison of average angles for different finger receptive areas in non-SDS situation.

	Sig. (Adj. Sig. *)	
	Region B	Region C
Region A	<0.001 (<0.001)	0.027 (0.081)
Region B	-	0.114 (0.342)

* Deviation values are significant at Sig. ≤ 0.05. Significance values have been adjusted by the Bonferroni correction for multiple tests.

## Data Availability

Data are contained within the article.
